# Aloe emodin relieves Ang II‐induced endothelial junction dysfunction via promoting ubiquitination mediated NLRP3 inflammasome inactivation

**DOI:** 10.1002/JLB.3MA0520-582R

**Published:** 2020-06-23

**Authors:** Yi Zhang, Ziqing Song, Shan Huang, Li Zhu, Tianyi Liu, Hongyan Shu, Lei Wang, Yi Huang, Yang Chen

**Affiliations:** ^1^ School of Pharmaceutical Guangzhou University of Chinese Medicine Guangzhou University Town Guangzhou China; ^2^ Department of Stomatology The First Affiliated Hospital, The School of Dental Medicine Jinan University Guangzhou China

**Keywords:** Aloe emodin, Ang II, endothelial dysfunction, NLRP3 inflammasome, NLRP3 ubiquitination

## Abstract

Recent studies have revealed that aloe emodin (AE), a natural compound from the root and rhizome of *Rheum palmatum* L., exhibits significant pharmacologic activities. However, the pharmacologic relevance of the compound, particularly for cardiovascular disease, remains largely unknown. Here, we hypothesized that AE could improve endothelial junction dysfunction through inhibiting the activation of NOD‐like receptor family pyrin domain containing‐3 (NLRP3) inflammasome regulated by NLRP3 ubiquitination, and ultimately prevent cardiovascular disease. In vivo, we used confocal microscopy to study the expression of tight junction proteins zonula occludens‐1/2 (ZO‐1/2) and the formation of NLRP3 inflammasome in coronary arteries of hypertension. And the experimental serum was used to detect the activation of NLRP3 inflammasome by ELISA assay. We found that AE could restore the expression of the endothelial connective proteins ZO‐1/2 and decrease the release of high mobility group box1 (HMGB1), and also inhibited the formation and activation of NLRP3 inflammasome. Similarly, in vitro, our findings demonstrated that AE could restore the expression of the tight junction proteins ZO‐1/2 and decrease monolayer cell permeability that related to endothelial function after stimulation by angiotensin II (Ang II) in microvascular endothelial cells (MECs). We also demonstrated that AE could inhibit Ang II‐induced NLRP3 inflammasome formation and activation, which were regulated by NLRP3 ubiquitination in MECs, as shown by fluorescence confocal microscopy and Western blot. Together with these changes, we revealed a new protection mechanism of AE that inhibited NLRP3 inflammasome activation and decreased the release of HMGB1 by promoting NLRP3 ubiquitination. Our findings implicated that AE exhibited immense potential and specific therapeutic value in hypertension‐related cardiovascular disease in the early stage and the development of innovative drugs.

## Graphical Abstract

Aloe Emodin improves endothelial junction dysfunction by inhibiting NLRP3 inflammasome activation through NLRP3 ubiquitination; may have specific therapeutic value in hypertension‐related cardiovascular disease.

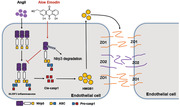

**Abbreviations**
AE: Aloe emodin
Ang II: angiotensin II
ASC: apoptosis‐associated speck‐like protein containing CARD
gRNA: Single‐guide RNA
HMGB1: high mobility group box1
IP: immunoprecipitation
MECs: Microvascular endothelial cells
NLRP3: NOD‐like receptor family pyrin domain containing‐3
RIPA: radio immunoprecipitation assay
ROS: reactive oxygen species
TEER: trans endothelial electric resistance
ZO‐1/2: zonula occludens‐1/2

## 1 INTRODUCTION

Cardiovascular disease is the primary cause of overall mortality throughout the world, and hypertension is one of the major factors. Increasing evidence shows that angiotensin II (Ang II) is a key mediator of hypertensive.^1^ When Ang II levels increase, oxidative stress occurs, which could increase blood pressure by several mechanisms.^2^ These include inadequate production or reduction of bioavailability of NO, alterations in metabolism of arachidonic acid, resulting in an increase in vasoconstrictors and decrease in vasodilators, and up‐regulation of endothelin.^3^ Ang II plays an important role in the initiation and progression of endothelial dysfunction, including Ang II‐mediated vascular tone dysfunction,^4^ Ang II‐ and endothelium‐mediated vascular inflammation,^5^ Ang II‐related atherosclerosis,^6^ and vascular remodeling.^7^ Endothelial dysfunction is a well‐established fundamental cause of cardiovascular diseases and also a predictor of worse clinical outcomes. Endothelial cell barrier dysfunction results in many pathologic consequences including endothelial cell injury reflecting in tight junction protein zonula occludens‐1/2 (ZO‐1/2), endothelium inflammation, and impairs endothelial integrity further.^8^ These interendothelial junction proteins play critical roles in protecting blood vessels and then maintain life stability. However, there are few effective drugs in clinical prevention and treatment of the endothelial injury caused by hypertension currently. As a consequence, it is imperative to find a drug that can maintain the homeostasis of endothelial tight junction proteins.

As a traditional Chinese medicine, Rheum has been widely and successfully used for many years. Aloe emodin (AE), 1,8‐Dihydroxy‐3‐(hydroxymethyl) anthraquinone, is a major anthraquinone derived from the root and rhizome of *Rheum palmatum* L. Clinical studies on AE has been demonstrated that it produces beneficial effects including antioxidant,^9^ anti‐angiogenic,^10^ anti‐metastasis,^11^ anti‐inflammatory,^12^ and anti‐cancer.^13^ It has been proven that AE could effectively prevent cardiovascular diseases.^14^ AE prevented high‐fat diet‐induced QT prolongation by repressing miR‐1 and up‐regulating its target Kir2.1.^14^ AE protected against myocardial infarction via the up‐regulation of miR‐133, suppression of caspase‐3 apoptotic signaling pathway.^15^ But the protective effect of AE against endothelial dysfunction in the pathogenesis of Ang II‐induced vascular complications and the underlying mechanism remains largely unknown.

It is reported that AE possessing antioxidant effect and alteration of reactive oxygen species (ROS) production may represent an important mechanism for AE mediated regulation of cellular behaviors.^15^ AE has been shown to prevent high glucose‐induced β‐cell toxicity via inhibition of ROS and down‐regulation of apoptosis.^16^ ROS is one of the pathways that activates NOD‐like receptor family pyrin domain containing‐3 ( NLRP3) inflammasome.^17^ The NLRP3 inflammasome monomer is a multi‐subunit protein, which consists of pattern recognition receptor NLRP3, apoptosis‐associated speck‐like protein containing CARD (ASC), and inactive pro‐caspase‐1. Pro‐caspase‐1 is converted to its active form, cle‐caspase‐1, and subsequently cleaves its substrates such as pro‐IL‐1β to bioactive IL‐1β. These canonical cytokine mediate inflammatory responses in many kinds of cells such as endothelial cells.^18^ So it could cause or exacerbate the endothelial inflammation contributing to the development of cardiovascular diseases. NLRP3 inflammasome contributes to the increased release of high mobility group box1 (HMGB1),^19^ one of the major damage‐associated molecular patterns (DAMPs). The protective mechanisms of other effective drugs have been shown to prevent endothelial dysfunction related to NLRP3 inflammasome, such as Asprin^20^ and puerarin.^21^ It has also been reported that AE possesses anti‐inflammatory effect. Hence, we hypothesized that the protective effect of AE on cardiovascular disease may be related to NLRP3 inflammasome.

In this study, our finding raised the possibility that AE confers protection against endothelial injury induced by Ang II in endothelium and reveals the important mechanism of NLRP3 inflammasome regulated by AE for the first time. Our study showed that AE protected the expression of interendothelial junction proteins and restricted the release of HMGB1 by inhibiting the activation of NLRP3 inflammasome regulated by NLRP3 ubiquitination, and ultimately prevented cardiovascular disease. Our study implicates the clinical potential of AE for the prevention of hypertension‐induced cardiovascular diseases in the early stage.

## 2 MATERIALS AND METHODS

### 2.1 Animal experiment

All experiments were done with C57BL/6J male mice age‐matched 8 wk old (20–25 g), and housed with water and food ad libitum in a 12/12 h reverse light/dark cycle (lights on at 8:00 AM and off at 8:00 PM). All mice were bred from breeding pairs from Nanjing Biomedical Research Institute (Nanjing, China). All protocols were approved by the Institutional Animal Care and Use Committee of Guangzhou University of Chinese Medicine (Guangzhou, China). The mice were housed in the pathogen‐free animal experiment room (temperature 25 ± 2°C, relative humidity 50% ± 5%; a 12/12 h light/dark cycle. 0.9% NaCl containing Ang II (1.44 mg/kg/d; D700, Sigma, Darmstadt, Germany) was administered by subcutaneous (s.c.) injection for three times per day to stimulate early hypertension with the vascular inflammation model. Forty‐eight male mice were randomly divided into six groups: Control group (*n *= 8), Ang II group (*n *= 8), AE low concentration group (*n *= 8), AE medium concentration group (*n *= 8), AE high concentration group (*n *= 8), and losartan group (*n *= 8). Mice were treated with 0.5% sodium carboxymethylcellulose (C5678, Sigma), Lorsartan (20 mg/kg; T0215L, TargetMol), or AE (10 mg/kg, 20 mg/kg, or 30 mg/kg; 481‐72‐1, Pufeide, Chengdu). The treatment with AE or losartan was combined with Ang II. After 3 wk, mice were humanely sacrificed after fasting for 12 h. Blood was centrifuged for 20 min at 3000 rpm and 4°C in refrigerate centrifuge (Sigma, 3K15), and plasma was collected. Heart tissue and plasma samples were kept at 80°C until analyzed for inflammatory markers. Blood pressure was measured by the tail sleeve in mice.

### 2.2 Cell culture

Microvascular endothelial cells (MECs) line hemangioendothelioma (EOMA) was purchased from ATCC (Shanghai, China). The cell line was originally isolated from mouse hemangioendothelioma. MECs were cultured in DMEM (Gibco, 11995‐065, Rockford, IL, USA), containing 10% of FBS (Gibco, 10099‐141C), and 1% penicillin‐streptomycin (Gibco, 15140‐122). The cells were cultured in a humidified incubator at a mixture at 37°C with 5% CO_2_ and 95% N_2_. Cells were passaged by trypsinization (0.25% Trypsin/EDTA; Gibco, 25200‐056), followed by dilution in DMEM medium containing 10% FBS. The cells were treated with five cases: control group, model group with Ang II (20 nM), and different concentrations AE group (0.05–0.5 µM). And then all cells, being seeded in 6‐well plates at a density 5 × 10^5^ cells/mL were incubated in DMEM medium with 10% FBS for 24 h.

### 2.3 Blood pressure measurement

Blood pressure was measured using tail‐cuff plethysmography (Softron Systems BP‐2010 *Series II Blood Pressure Analysis System*) including systolic blood pressure and diastolic blood pressure. Briefly, mice were trained for a period of five consecutive days in which blood pressure was measured (30 measurements per day) but not recorded. Following this training period, blood pressure was measured for another three consecutive days, recorded, and averaged.

### 2.4 Immunoblotting

MECs were washed twice with ice‐cold PBS and scraped in radio immunoprecipitation assay (RIPA) buffer (Thermo Scientific, Rockford, MI, USA) containing a protease inhibitor cocktail (Roche, 04693132001, Basel, Switzerland). After centrifugation at 12000 ×*g* for 10 min at 4°C, the supernatants containing the membrane protein and cytosolic components, termed homogenates, were frozen and store at −20°C until use. The cell supernatants were tested by BCA protein assay kit (Beyotime, Beijing, China) and denatured with 5× protein loading buffer (SDS‐PAGE; Beyotime) in a metal bath for 5 min, followed by cooling on ice for another 5 min. Samples were run on 12% SDS‐PAGE and transferred onto 0.22 µm polyvinylidene fluoride membranes and blocked with 5% nonfat milk for 2 h at room temperature. The membranes were probed with indicated primary antibodies at 4°C overnight followed by incubation with anti‐rabbit IgG (1:2000; Cell Signaling Technology, Danvers, MA, USA) or anti‐mouse IgG (1:2000; CST) for 1 h at room temperature. The primary antibodies were anti‐NLRP3 (1:1000; CST), anti‐caspase‐1 (8:5000; Santa Cruz, Dallas, TX, USA), anti‐Ubiquitin (1:1000; Abcam, Cambridge, MA, USA), and anti‐ASC (8:5000; Santa Cruz). The anti‐beta‐actin (1:1000; CST) was used as an internal control. Protein bands were visualized using the Western blotting detection system (Tanon, Shanghai, China) according to the manufacturer's instructions and analyzed using Image J software (NIH, Bethesda, USA).

### 2.5 Immunofluorescence microscopy analysis

To detect inflammasome activation in EOMA and the endothelium of mouse coronary arteries, we carried out immunofluorescence. Cells grown on culture dish at a density of 5 × 10^5^ cells/mL were treated for 24 h and then fixed in 4% paraformaldehyde for 15 min after PBS washing for three times. The frozen slides from heart tissues were fixed in acetone for 15 min, then incubated overnight at 4°C with primary antibody. After incubation with primary antibodies, the dishes or slides were washed and labeled with corresponding Alexa Fluor‐488 and Alexa Fluor‐555 conjugated secondary antibodies (Invitrogen, Carlsbad, CA, United States) co‐incubated for 1.5 h at room temperature. Then, the dishes or slides were washed, mounted, and visualized through sequentially scanning on a laser scanning confocal fluorescence microscope (Carl Zeiss, Oberkochen, Germany). Colocalization in vein or cells were analyzed by Image Pro Plus software (Media Cybernetics, Rockville, MD, USA), and the colocalization coefficient was represented by Pearson's correlation coefficient. Goat anti‐NLRP3 antibody (1:200, Abcam), mouse anti‐caspase‐1 antibody (1:200; Santa Cruz), rabbit anti‐ASC antibody (1:200; Santa Cruz), anti‐ZO‐1 (1:500, Invitrogen, Grand Island, NY, USA), rabbit anti‐ZO‐2 antibody (1:250; Invitrogen, Carlsbad, CA, United States), rabbit anti‐HMGB1 antibody (1:200; Santa Cruz), and mouse anti‐PECAM antibody (1:200; Santa Cruz) were used for these experiments.

### 2.6 ELISA assay for cytokine

After treatment, the cell supernatant and mice serum were collected and HMGB1 and IL‐1β production were measured by a commercially available ELISA kit (R&D System, Minneapolis, MN, USA) according to the protocol described by the manufacturer.

### 2.7 Endothelial permeability measurement

Endothelial permeability was designed by the measurement of FITC‐dextran flux across monolayers of cultured endothelial cells. Cells were plated on top of transwell chambers in 24‐well plates (0.4 µm pore size) and grown to confluence. The same stimulation and drug interference were given after the cells adhered to the wall. After FITC‐dextran 40 kDa (Sigma) was added into the upper chamber for 1 h, paracellular flux was assessed by taking 100 µL aliquots from both chambers to measure real‐time changes of permeability across endothelial cell monolayers. Fluorescence was detected in appointed samples using a fluorescent plate reader and emission wavelengths of 485 and 530 nm, respectively.

### 2.8 Determination of trans endothelial electric resistance (TEER)

TEER was performed by using an assembly containing current‐passing and voltage‐ measuring electrodes (EVOMZ, World Precision Instruments (WPI) is located at Sarasota, FL, USA). Cell treating is the same as steps in measuring cell permeability. MECs were also seeded on top of the transwell chamber in 24 wells (0.4 µm pore size).

### 2.9 Single‐guide RNA (gRNA) transfection

NLRP3 gene was knocked down in Carotid arterial endothelial cell (CAEC) by gRNA, which targeted steady endothelial gNLRP3 expression. gRNA sequences for CRISPR/Cas9 gene editing of coding genes were projected by the CRISPR Design tool (http://crispr.mit.edul). gRNA sequences were synthesized and then inserted into the Bbsl‐digested PX459 plasmid. gNLRP3 sequences were 5′‐GACGAGTGTCCGTTGCAAGC‐3′. Gene editing in CAECs was used in lipofectamine 3000 transfection kit (Invitrogen, Carlsbad, CA, USA) according to manufacturer's guidelines. The transfected cells were incubated in the medium with 2.5 µg/ml puromycin to screen out the gRNA cells that contained plasmid.

### 2.10 Immunoprecipitation (IP)

For IP assays, cells were treated and incubated for 24 h. Cells were lysed in RIPA buffer (Thermo Scientific) containing a protease inhibitor cocktail (Roche, 04693132001). After centrifugation at 12000 ×*g* for 10 min at 4°C, the cell supernatants were blocked with protein G PLUS‐agarose (Santa Cruz, sc‐2002) for 30 min. For NLRP3 IP analyses, each supernate containing 500 µg total protein was incubated with the anti‐NLRP3 antibody (D4D8T, CST) at 4°C overnight. On the next day, 20 µL protein G beads (Santa Cruz Biotechnology and Santa Cruz are the same manufacturers) were added into each lysate at 4°C shaking cultivation for 3.5 h. The beads were washed three times with lysis buffer and heated at 98°C for 5 min in 40 µL lysis buffer and 10 µL 5× loading buffer. And then the precipitated proteins were subjected to immunoblotting analysis. To validate the levels of ubiquitination of NLRP3 in MECs after AE treatment induced by Ang II, we performed ubiquitination (ab7780, Abcam).

### 2.11 Statistical analysis

All results were showed as mean ± sem of three to eight independent experiments with each experiment including triplicate sets. One‐way ANOVA analysis and post hoc tests were applied when there were more than two groups in the independent variable. The level of significance was set at a *P*‐value of 0.05.

## 3 RESULT

### 3.1 Aloe emodin suppressed Ang II‐induced disruption of tight junction proteins in coronary arterial endothelium and blood pressure abnormity

**FIGURE 1:**
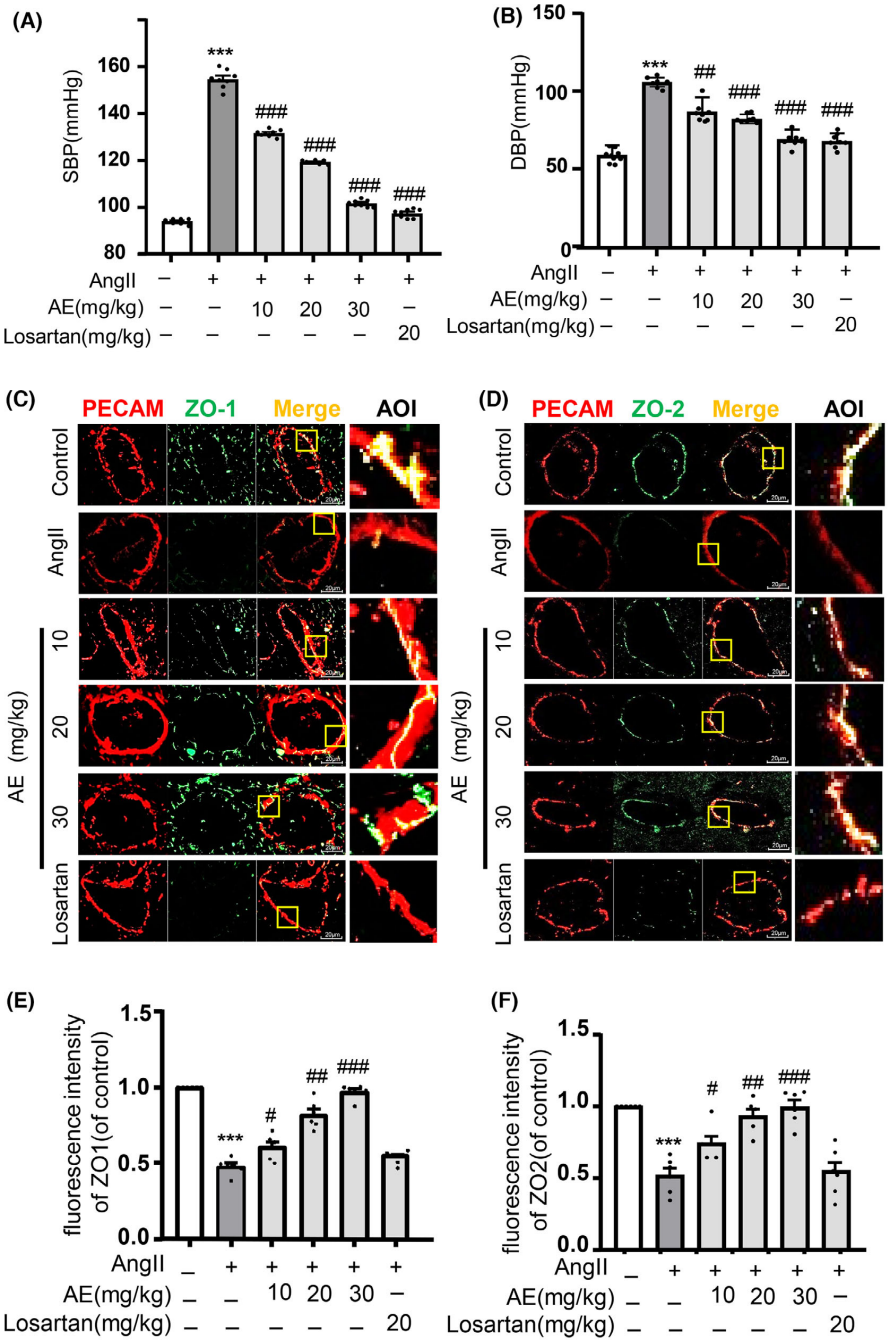
**Aloe emodin (AE) protected the integrity of the endothelium and restored blood pressure in vivo**. Blood pressure was measured by tail sleeve in mice. (**A**) and (**B**) Summarized data showing the systolic blood pressure and diastolic blood pressure (*n *= 8). (**C**) and (**D**): Zonula occludens‐1 (ZO‐1)/PECAM and ZO‐2/PECAM were identified by confocal microscopy with frozen sections of mouse hearts, the merged images displayed yellow dots or patches indicating the colocalization of ZO‐1 or ZO‐2 (green) with PECAM (red). (**E**) Summarized data showing the colocalization coefficient of ZO‐1 with PECAM (*n *= 6). (**F**) Summarized data showing the colocalization coefficient of ZO‐2 with PECAM (*n *= 6). ^***^ *P *< 0.001, angiotensin II (Ang II) vs. Control, ^#^ *P *< 0.05, ^##^ *P *< 0.01, ^###^ *P *< 0.001, AE/Losarten vs. Ang II. Scale bar, 20 µM

Mice were fed normal chow diet for 8 wk, and the experiment lasted for 3 wk. After Ang II (1.44 mg/kg/d) with 0.9% normal saline was administered by s.c. injection for three times per day to stimulate early hypertension with the vascular inflammation model. To determine the success of the vascular endothelial injury and early hypertension model, the damage of endothelial function was determined by examining the changes in the gap protein ZO‐1/2 and the blood pressure was measured at the same time. As shown in Fig. 1, we found that AE recovered the level of systolic pressure, diastolic pressure, and expression ZO‐1/2 after Ang II stimulation. In other words, AE could repair blood pressure and endodermal permeability.

### 3.2 Aloe emodin alleviated Ang II‐induced inflammasome activation and HMGB1 release in coronary arterial endothelium

**FIGURE 2:**
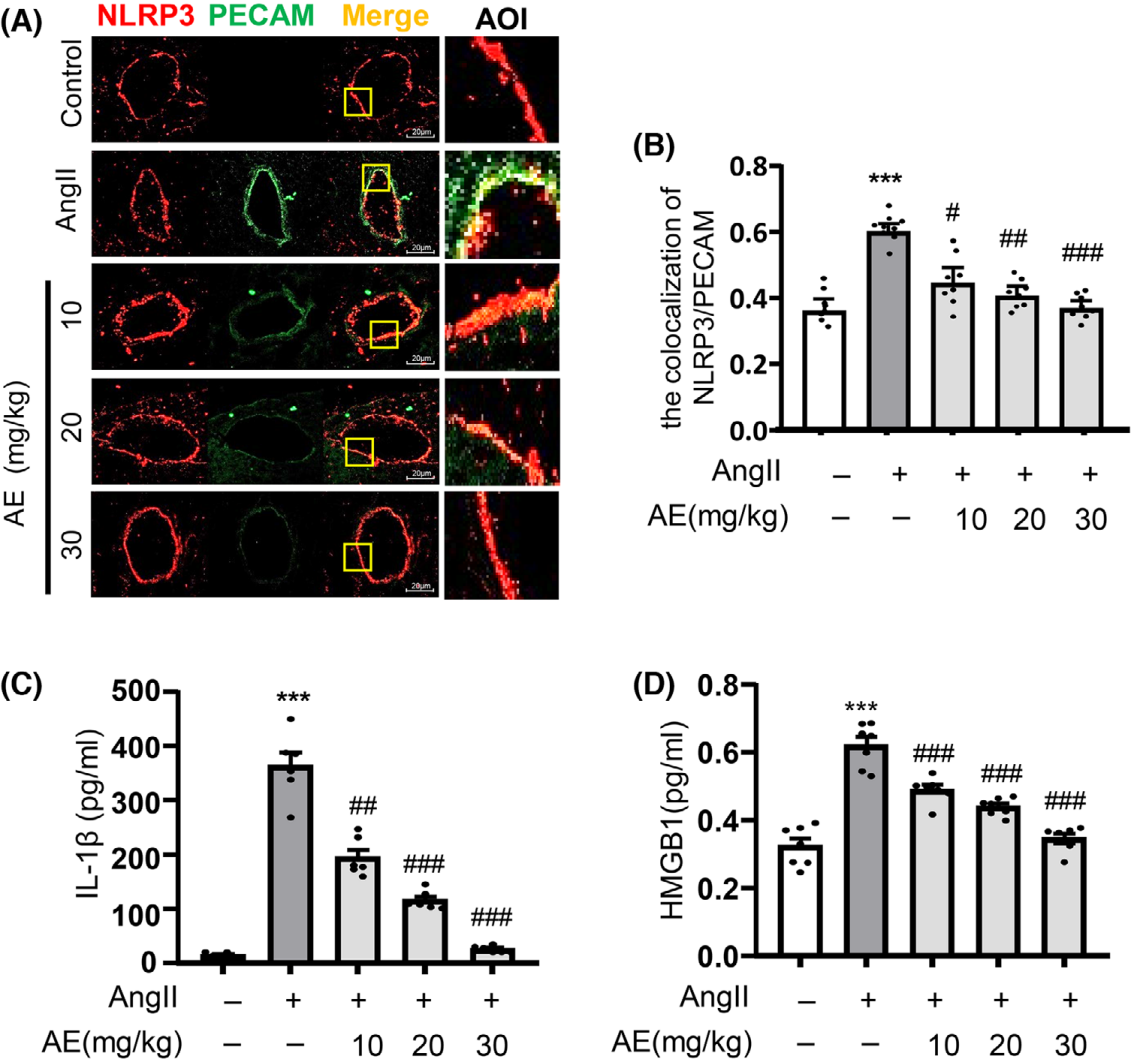
**Aloe emodin (AE) suppressed angiotensin II (Ang II)‐induced activation of NOD‐like receptor family pyrin domain containing‐3 (NLRP3) inflammasome and high mobility group box1 (HMGB‐1) expression in vivo**. (**A**) Frozen sections of mouse hearts were stained for PECAM, and Alexa 488‐conjugated antibodies against NLRP3 in coronary arteries. The merged images displayed yellow dots or patches indicating the colocalization of PECAM (red) with NLRP3 (green). (**B**) Summarized data showing the colocalization coefficient of PECAM with NLRP3 (*n *= 8). (**C**) and (**D**): The content of IL‐1β (*n *= 6) and HMGB1 (*n *= 7) in serum were detected by ELISA kit. ^***^ *P *< 0.001, Ang II vs. Control, ^#^ *P *< 0.05, ^##^ *P *< 0.01, ^###^ *P *< 0.001, and AE vs. Ang II. Scale bar, 20 µm

Through colocalizing NLRP3 with PECAM by immunofluorescence, we found that AE inhibited the activation of NLRP3 inflammasome (Fig. 2A, B). The activation of inflammasome reflecting in IL‐1β expression of plasma in each group after stimulation and administration were shown in Fig. 2C. Similarly, we found that AE inhibited the activation of inflammation in coronary arterial endothelium. When serum HMGB1 was detected by ELISA, the release of HMGB1 was decreased after AE treatment (Fig. 2D).

### 3.3 Aloe emodin prevented micro‐endothelial dysfunction induced by Ang II in MECs

**FIGURE 3:**
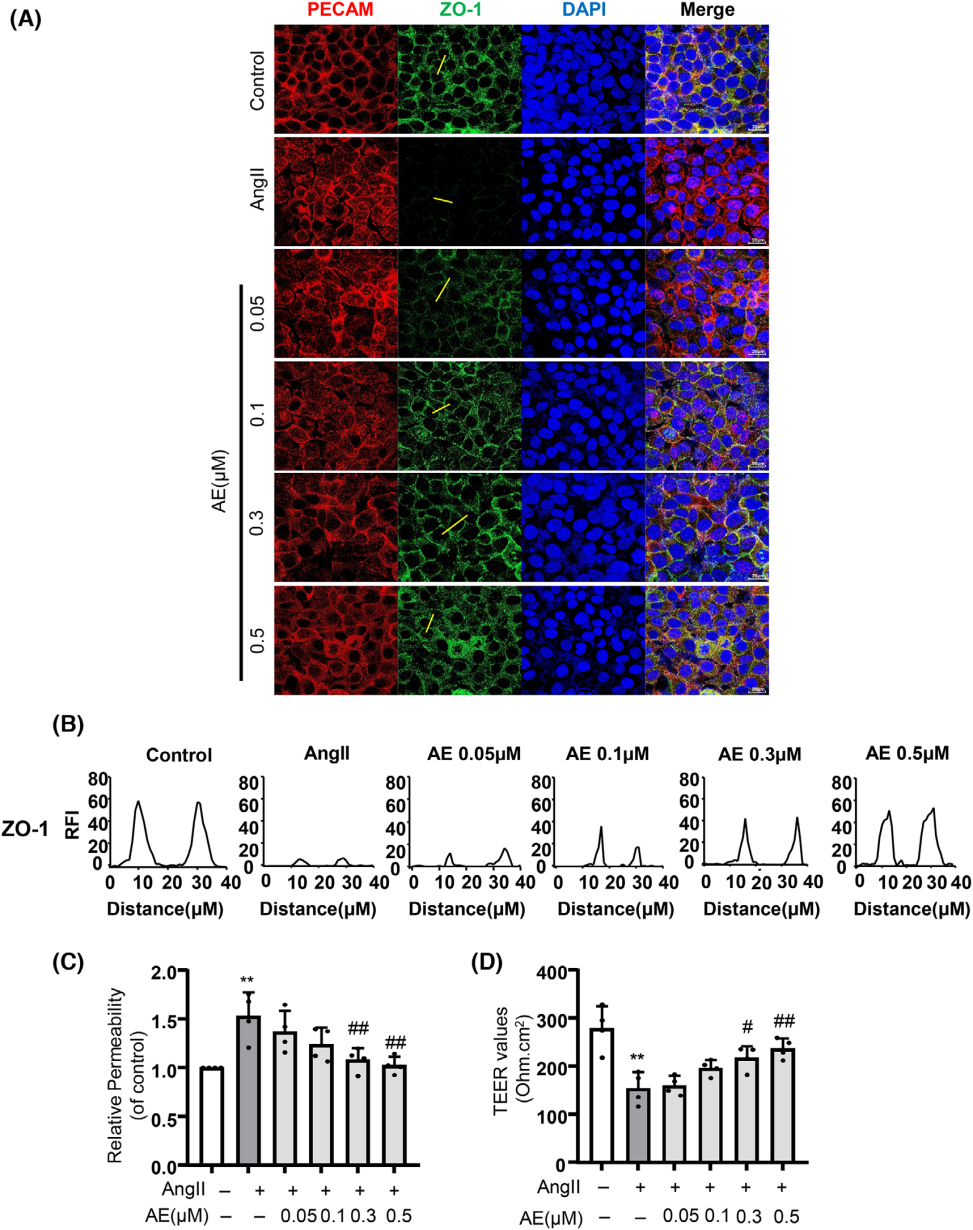
**Aloe emodin (AE) repaired the integrity of the endothelium**. (**A**) and (**B**) The expression of zonula occludens‐1 (green) in endothelial cells was detected by fluorescence microscopy to determine the integrity of endothelial cells (*n *= 4). Cells were co‐immunostained with anti‐PECAM (red) and DAPI (blue). (**C**) The integrity of endothelial cells permeability (*n *= 4). (**D**) The integrity of endothelial cells was determined by detecting changes in trans endothelial electric resistance (*n* = 4). ^**^ *P *< 0.01, angiotensin II (Ang II) vs. Control, ^#^ *P *< 0.05, ^##^ *P *< 0.01, AE vs. Ang II. Scale bar, 20 µm

Tight junction proteins and adhesion junction proteins play a critical role in mediating the permeability of solutes between adjacent endothelium cells. Disruption of junction proteins increases endothelial cells permeability during vascular dysfunction. As shown in Fig. 3A and B, MECs stimulated with Ang II markedly decreased the expression of tight junction proteins ZO‐1, which was obviously restored by AE. The results of the endothelial permeability and TEER are consistent with the expression of ZO‐1 (Fig. 3C, D), which proved that AE may have an effect of protecting the tight junction proteins for endothelium.

### 3.4 Aloe emodin inhibited the formation and activation of NLRP3 inflammasome and HMGB1 expression in MECs with Ang II stimulation

**FIGURE 4:**
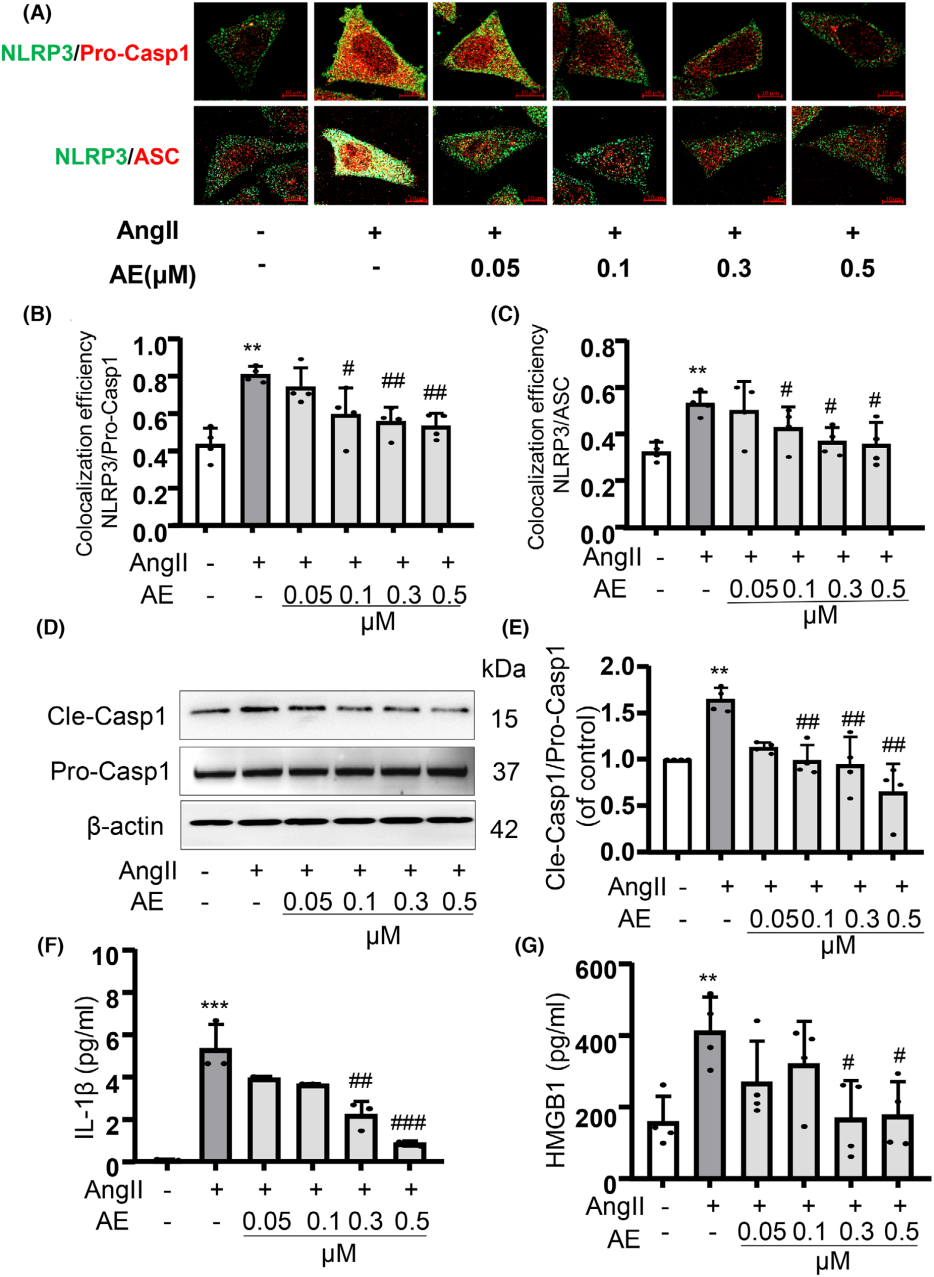
**Aloe emodin (AE) inhibited the recruitment and activation of NOD‐like receptor family pyrin domain containing‐3 (NLRP3) inflammasome and high mobility group box1 (HMGB1) expression**. (**A**), (**B**), and (**C**): NLRP3/caspase‐1 and NLRP3/ASC were identified by confocal microscopy to determine the formation of the inflammasome (*n* = 4). (**D**) and (**E**): Western blot analysis of pro‐caspase‐1 and cle‐caspase‐1 expression (*n *= 4). (**F**) and (**G**): The content of IL‐1β (*n *= 3) and HMGB1 (*n *= 4) in cell supernatant were detected by ELISA kit. ^**^ *P *< 0.01, ^***^ *P *< 0.001, angiotensin II (Ang II) vs. Control, ^#^ *P *< 0.05, ^##^ *P *< 0.01, ^###^ *P *< 0.001, AE vs. Ang II. Scale bar, 10 µm

The NLRP3 inflammasome is best characterized as a type of inflammasome in mammalian cells that consists of a proteolytic complex formed by NLRP3, ASC, and pro‐caspase‐1. To biochemically assess the anti‐inflammatory reduction effect of AE, we detected NLRP3 inflammasome protein expression and inflammasome formation. The immunofluorescence colocalization method was used to detect the recruitment of inflammasome (Fig. 4A–C). Moreover, AE significantly inhibited the recruitment of NLRP3 inflammasome. To confirm the activation of NLRP3 inflammasome, we detected caspase‐1 inflammasome protein expression, containing pro‐caspase‐1, which relates to the recruitment of NLRP3 inflammasome, and cle‐caspase‐1, which relates to the activation of NLRP3 inflammasomes (Fig. 4D, E). AE clearly decreased cleavage caspase‐1 in Ang II‐treated MECs. NLRP3 inflammasome is activated to produce a number of inflammasome products including the HMGB1 and IL‐1β, which serve as a novel vascular permeability factor to mediate vascular hyper‐permeability and promote endothelial cell‐mediated vascular remodeling. As shown in Fig. 4F and G, we found that the release of IL‐1β and HMGB1 were increased with the stimulation of Ang II and the release level was decreased after AE intervention.

### 3.5 Aloe emodin abolished Ang II‐induced disassembly of tight junction protein via NLRP3 pathway in MECs

**FIGURE 5:**
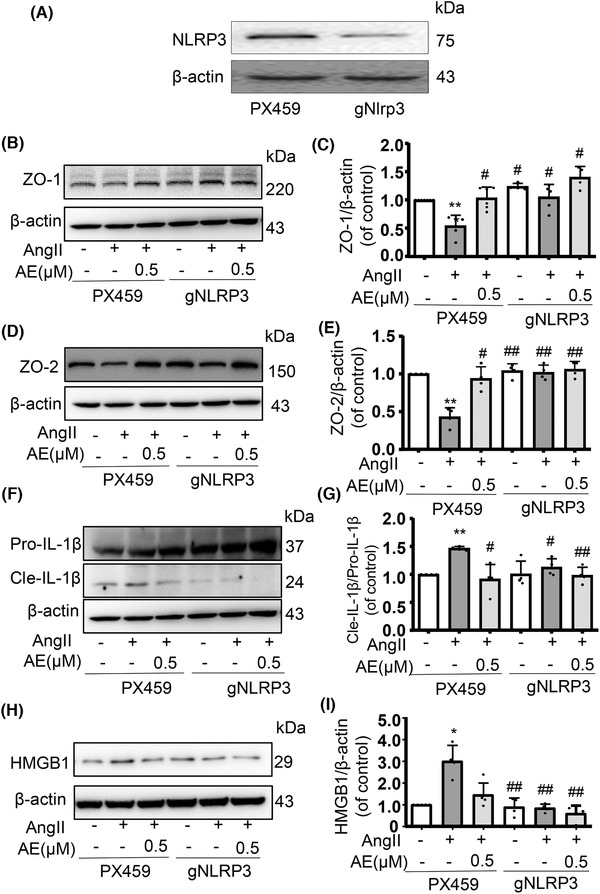
**Aloe emodin (AE) had no therapeutic effect on gRNA NOD‐like receptor family pyrin domain containing‐3 (NLRP3) cells**. AE was determined to protect endothelial integrity by inhibiting NLRP3 inflammasome. (**A**) NLRP3 gene knockdown succeeded. (**B**) and (**C**) and (**D**) and (**E**): Detecting the expression of zonula occludens‐1 (ZO‐1) and ZO‐2 in gNLRP3 endothelial cells by Western blot analysis, the expression was unchanged regardless stimulation or administration (*n *= 4). (**F**) and (**G**): Western blot analysis of Pro‐IL‐1β and Cle‐IL‐1β expression in cells (*n *= 4). (**H**) and (**I**) Western blot analysis of high mobility group box1 expression in cells (*n *= 4). ^*^ *P *< 0.05, ^**^ *P *< 0.01, angiotensin II (Ang II) of PX459 vs. Control of PX459, ^#^ *P *< 0.05, ^##^ *P *< 0.01, AE of PX459/gNLRP3 vs. Ang II of PX459

To prove that AE could protect the endothelial function by inhibiting NLRP3 inflammasome, the gene of NLRP3 was knocked down in MECs gRNA and MECs stably expressing gNLRP3 were established. The silence efficiency was shown by Western blot analysis (Fig. 5A). Compared with the scramble group, the Ang II stimulation of the knockdown group showed no obvious inhibition on the expression of ZO‐1/2, IL‐1β, HMGB1 protein, and there was no significant change after AE administration (Fig. 5B–I).

### 3.6 Aloe emodin restrained activation of NLRP3 inflammasome is associated with NLRP3 ubiquitination

**FIGURE 6:**
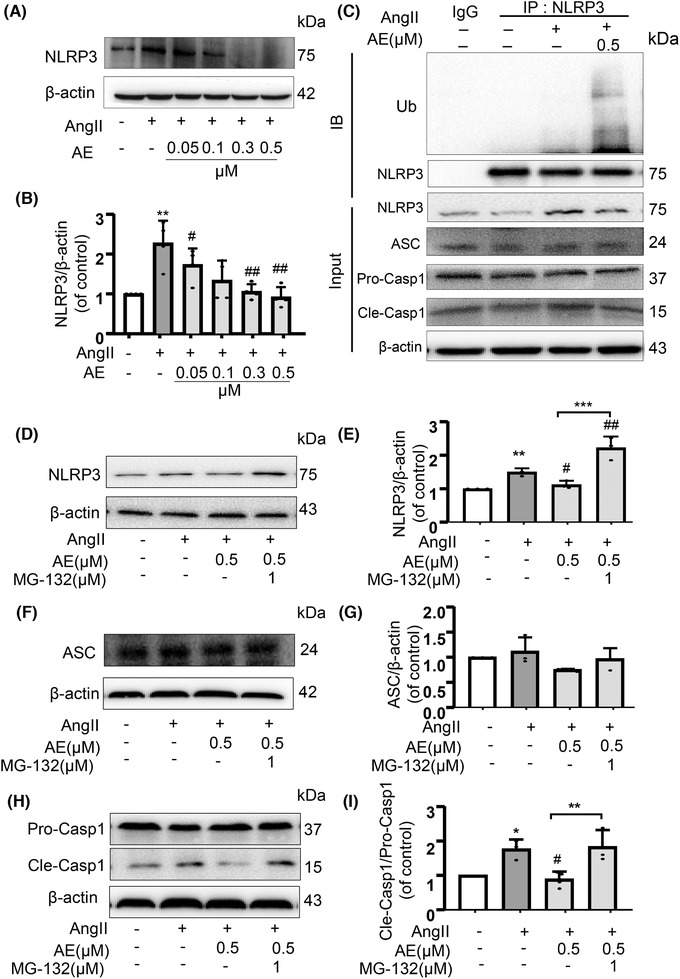
**(A) and (B) Western blot analysis of NOD‐like receptor family pyrin domain containing‐3 (NLRP3) expression in different aloe emodin (AE) concentrations (0.05, 0.1, 0.3, 0.5 µmol/L) after angiotensin II (Ang II)‐induced (*n* = 4)**. (**C**) Cell lysates were immunoprecipitated with control and IgG or anti‐NLRP3 Ab and analyzed by immunoblotting with anti‐ubiquitin (Ub). Ub and NLRP3 related protein from total cell lysates were analyzed by Western blot. (**D**) and (**E**): Western blot analysis of NLRP3 expression treated with MG‐132 (*n *= 3). (**F**) and (**G**): Western blot analysis of ASC expression treated with MG‐132 (*n *= 3). (**H**) and (**I**): Western blot analysis of pro‐caspase‐1 and cle‐caspase‐1 expression treated with MG‐132 (*n *= 3). ^*^ *P *< 0.05, ^**^ *P *< 0.01, ^***^ *P *< 0.001, Ang II vs. Control, ^#^ *P *< 0.05, ^##^ *P *< 0.01, AE/AE with MG‐132 vs. AE group or AE with MG‐132 group vs. Ang II

We detected NLRP3 inflammasome protein expression with Ang II stimulia. NLRP3 inflammasome protein expression (Fig. 6A, B) was tremendously reversed after AE treatment. AE treatment dramatically down‐regulated the expression of NLRP3. Next, we asked how AE signaling inhibits NLRP3 inflammasome activation. We then guessed whether AE mediated the degradation of ubiquitinated NLRP3 protein. Consistent with our hypothesis, treatment with AE after Ang II stimulation induced ubiquitination of NLRP3 in MECs (Fig. 6C). Importantly, AE‐induced NLRP3 degradation was also restored when treating with MG‐132 in MECs (Fig. 6D, E). Subsequently, MG‐132 treatment promoted NLRP3 inflammasome activation protein, such as the expression of cle‐caspase‐1, but no change in pro‐caspase‐1 expression and ASC expression (Fig. 6F–I). Our results showed that proteasome inhibitor MG‐132 could suppress AE‐induced NLRP3 degradation, suggesting that ubiquitinated NLRP3 is upgraded by AE.

## 4 DISCUSSION

Here, we provide the evidence for a new therapeutic application of AE to protect against endothelial injury in cardiovascular diseases. To our knowledge, this is a new theory that AE restricts the activation of NLRP3 inflammasome in endothelium through the ubiquitination of NLRP3.

MECs are connected to each other by a complex set of junctional proteins, including tight junction (ZO‐1/2). These endothelial tight junction proteins play critical roles in protecting cardiovascular diseases in the early stage and then maintain life stability. In our study, we found that Ang II could significantly decrease the expression of tight junction protein ZO‐1/2, which means that Ang II could contribute to endothelial cell injury. Other studies also reported that Ang II involved in the development of various cardiovascular diseases by disrupting microvessel permeability,^22^ inhibited the protein expression of ZO‑1 in vascular endothelial cells by down‐regulating vascular endothelial (VE)‑cadherin, and then destroying the tight junctions between endothelial cells.^23^ Therefore, the prevention and treatment of endothelial dysfunction induced by Ang II should be taken into account. In addition, the results of this study showed that the increase in blood pressure caused by Ang II was normalized by treatment with an AT‐1 receptor inhibitor losartan remarkably, and AE exerted the similar protective effect (Fig. 1A, B), demonstrating that AE is an effective treatment to hypertension‐related cardiovascular diseases. Our study also explored that AE could repair the integrity of the endothelial cytomembrane under the Ang II stimulations could prevent and cure endothelial dysfunction. It is reported that AE treatment could significantly reduce infarct size, and ameliorate impaired cardiac function.^15^ AE could be used to alleviate cerebral ischemia reperfusion injury.^24^ Together with the findings and our experiment, AE application should be considered as a simple and inexpensive prophylactic measure for cardiovascular event prevention in the early stage. Our study showed that losartan lacks the capacity to normalized junctional dysfunction in ECs, but restores hypertension. Because losartan prevents hypertension through other mechanisms, including promoting vasodilation and antagonizing the vasoconstrictor actions like arachidonic acid. However, the cellular mechanism of AE in modulating endotheliocyte functions remains largely unknown. So it is meaningful to search the mechanism of AE.

To further explore the mechanism, we focus on how AE regulated the tight junction protein ZO‐1/2. Currently, it has been demonstrated that the NLRP3 inflammasome plays an important role in the endothelial cell barrier dysfunction.^25^ And Ang II could cause a nonclassic inflammation response in the vascular endothelium and contribute to the inflammation response through the aggregation of intracellular ROS, which plays a critical role in NLRP3 inflammasome activation.^26^ As the data showed, we demonstrated that AE alleviated the Ang II‐induced vascular endothelial damage by inhibiting the activation of NLRP3 inflammasome in vivo (Fig. 2). Other studies also indicated that Ang II‐induced lung fibroblast α‐collagen I synthesis via NLRP3 inflammasome activation.^27^ And N‐acetylcysteine (NAC), Diphenyleneiodonium (DPI), or NADPH oxidases (NOX) depletion substantially suppressed the expression of Ang II‐induced NLRP3 inflammasome components.^28^ Therefore, the present study explored that AE is involved in inhibiting Ang II‐induced endothelial NLRP3 inflammasome formation and activation. Previous studies have shown that activation of NLRP3 inflammasome by injurious dangerous factor caused HMGB1 release and disruption of interendothelial junctions in cultured MECs.^19^ At the same time, our data confirmed that AE could significantly inhibit the release of HMGB1 in serum, which means that AE could significantly inhibit the activation of inflammasome. Our previous study has shown that NLRP3 inflammasome resulted in the disruption of endothelial tight junction and endothelial permeability.^25^ In the present work, our data confirmed that AE decreased NLRP3 inflammasome activation and HMGB1 release in vivo. We also found that the AE has a significant effect in vitro on restoring the endothelial permeability and junction protein ZO‐1/2 (Fig. 3). And we confirmed that AE could effectively inhibit the Ang II‐induced NLRP3 inflammasome formation and activation (Fig. 4). The results of in vitro and in vivo experiments showed that AE could restore endothelial tight junction proteins ZO‐1/2 and endothelial permeability via inhibiting the formation and activation of NLRP3 inflammasome. It means AE is effective in protecting vascular endothelium.

Straight after the shallow relationship between AE and NLRP3 inflammasome, we intended to further confirm the role of NLRP3 inflammasome on Ang II‐induced endothelial dysfunction in AE group. The reduced capacity of endothelial cells transfected with NLRP3 shRNA to release HMGB1 is consistent with recent findings that NLRP3 inflammasome activation could lead to translocation and secretion of HMGB1 by immune cells.^19^ Thus, we guessed that AE may recover endothelial tight junction disruption by inhibiting endothelial NLRP3 inflammasome activation and release of HMGB1. In our experiment, we knocked down the NLRP3 gene and detected the junction protein, IL‐1β, and HMGB1 (Fig. 5A). We demonstrated that AE inhibited the NLRP3 signaling pathway to alleviate endothelial dysfunction because ZO‐1/2 of gNLRP3 cells has no significant change in the normal group, model group, or administration group (Fig. 5B–E). In cultured endothelial cells, Ang II induced IL‐1β activation and HMGB1 production, which were blocked by NLRP3 gene silencing (Fig. 5F–I). To sum up, AE was verified that it relieved Ang II‐induced endothelial junction dysfunction, which is related to NLRP3 inflammasome signaling pathway.

At the same time, we found an interesting phenomenon that AE treatment dramatically down‐regulated the expression of NLRP3 in endothelium induced by Ang II (Fig. 6A, B). As we know, NLRP3 protein plays a pivotal role in NLRP3 inflammasome formation. Several mechanisms about inhibiting the NLRP3 protein have been reported. For example, BCL6 suppresses the NLRP3 transcription through binding to the NLRP3 promoter.^29^ TRIM31 as an E3 ubiquitin ligase, accelerates the degradation of NLRP3 via promoting K48‐linked polyubiquitination.^30^ Pellino2, which is dependent on Pellino2 forkhead‐associated (FHA) and really interesting new gene (RING)‐like domains, facilitates NLRP3 ubiquitination.^31^ These data highlight the fact that ubiquitination is a pivotal mechanism of suppressing NLRP3 inflammasome activation. Consequently, we further hypothesized that AE suppresses the NLRP3 inflammasome by increasing the ubiquitination of NLRP3, resulting in the good protection of endothelial cells. Our experiment also demonstrated that AE facilitated the ubiquitination of NLRP3 to inhibit activation of the NLRP3 inflammasome whereas the Ang II had no effect on the NLRP3 ubiquitination (Fig. 6C). Subsequently, the expression of NLRP3 and the activated caspase‐1 were reversed with the proteasome inhibitor MG‐132 treatment compared with the AE treatment (Fig. 6D, E, H, I). In conclusion, AE relieves Ang II‐induced NLRP3 inflammasome via promoting NLRP3 ubiquitination.

## 5 CONCLUSION

AE was explored to exert protective effects on hypertension‐related cardiovascular disease. The present results reveal a new protection mechanism of AE that inhibits NLRP3 inflammasome activation and decreases the release of HMGB1 by promoting NLRP3 ubiquitination, and thus restoring the endothelial tight junction proteins and permeability. Our findings indicate that AE exhibits immense potential therapeutic value in hypertension‐related cardiovascular disease and the development of an innovative drug.

## AUTHOR CONTRIBUTIONS

Yi Zhang, Ziqing Song and Shan Huang contributed to the animal experiments, cell experiments and data analysis. Li Zhu and Tianyi Liu contributed to the study analysis and data analysis. Hongyan Shu and Lei Wang contributed to the revision of the manuscript. Yi Huang and Yang Chen contributed to the conception and design of the project.

## DISCLOSURES

The authors declare no conflicts of interest.
